# Synthesis and Biological Activity of New 1,3-Dioxolanes as Potential Antibacterial and Antifungal Compounds

**DOI:** 10.3390/molecules16086806

**Published:** 2011-08-10

**Authors:** Hatice Başpınar Küçük, Ayşe Yusufoğlu, Emel Mataracı, Sibel Döşler

**Affiliations:** 1 Department of Chemistry, Faculty of Engineering, Istanbul University, 34320 Avcılar, Istanbul, Turkey; 2 Department of Pharmaceutical Microbiology, Faculty of Pharmacy, Istanbul University,34116 Bayezit, Istanbul, Turkey

**Keywords:** 1,3-dioxolanes, montmorillonite K10, diols, antibacterial activity, antifungal activity

## Abstract

A series of new enantiomerically pure and racemic 1,3-dioxolanes **1**-**8** was synthesized in good yields and short reaction times by the reaction of salicylaldehyde with commercially available diols using a catalytic amount of Mont K10. Elemental analysis and spectroscopic characterization established the structure of all the newly synthesized compounds. These compounds were tested for their possible antibacterial and antifungal activity. Biological screening showed that all the tested compounds, except **1**, show excellent antifungal activity against *C. albicans*, while most of the compounds have also shown significant antibacterial activity against *S. aureus*, *S. epidermidis*, *E. faecalis* and *P. aeruginosa*.

## 1. Introduction

1,3-dioxolanes are widely used in natural product syntheses as protecting groups for ketones, aldehydes and 1,2-diols and represent important intermediates and end-products in the pharmaceutical, fragnance and polymer industries [1,2,3]. Many biologically active compounds containing 1,3-dioxolane structure have been synthesized before. Aryl, alkyl, imidazole, triazole, pyrazole, benzimidazole, benzotriazole, oxypurine, pyrimidinyl and naphtyl groups are linked to 1,3-dioxolane ring at different positions (2,4 or 5). Depending on the structure of the substituents, these compounds exhibit a broad spectrum of biological activities such as antifungal [4], antibacterial [5,6], antineoplastic [7], antiviral [8,9], anesthetic [10,11] and anticonvulstant ones [12].

According to the literature survey, several 1,3-dioxolanes have been prepared using benzaldehyde and various aromatic aldehydes in the presence of acid catalyst [13,14,15]. 2,2'-bis(Hydroxymethyl) norborn-5-ene and ethylene glycol were used as 1,2-diol functionalities. We have recently reported [16] the synthesis of chiral and racemic 1,3-dioxolanes with aryl, mono substituted aryl and long alkyl chain groups (C_11_-C_13_).

The work reported herein was aimed at the preparation of new chiral and racemic 1,3-dioxolane derivatives with possible biological activities. It has been found that these new 1,3-dioxolanes with different substituents that have ether or ester groups at 3 and 4 positions displayed significant bacterial and fungicidal activities against certain bacteria. In the pharmaceutical industry, there has been an ever-increasing trend for chiral drug substances in enantiomerically pure form instead of racemic mixtures. As exemplified in the case of TADDOL [17], the absolute configuration of pharmaceuticals carries great importance for their biological activity. Therefore, it has been the intent in this work to examine the differences in biological activity between the enantiomeric and racemic structures.

Here, we would like to show the direct synthesis and biological activity, as antibacterial and antifungal agents, of some novel derivatives of chiral and racemic 1,3-dioxolanes produced in high isolated yield by the reaction of salicylaldehyde with chiral and racemic diols. Five different chiral (4*R*,5*R*)-1,3-dioxolanes (compounds **1**,**2**,**4**,**5**,**7**) were synthesized for the first time in this study (Table 1). The corresponding racemic dioxolane compounds **3**,**6**,**8** have not been found in the literature, therefore they were obtained as original racemic dioxolanes by acetalization reactions of the salicylaldehyde mentioned above with diols c, f and h (Table 1). 

**Figure 1:**
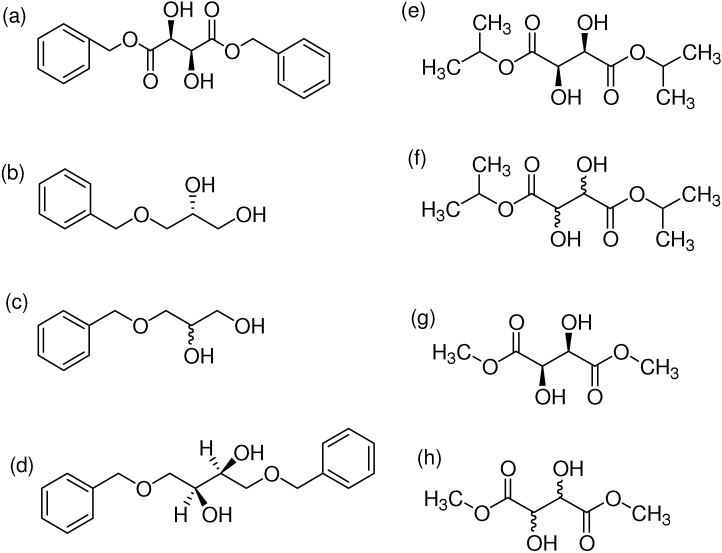
(a) (+)-Dibenzyl-L-tartrate; (b) (*R*)-(+)-3-Benzyloxy-1,2-propanediol; (c) (±)-3-Benzyloxy-1,2-propanediol; (d) (−)-1,4-Di-*O*-benzyl-L-threitol; (e) (*R,R*)-Diisopropyl-L-tartrate, (f) (±) Diisopropyl tartrate, (g) (*R,R*)-Dimethyl-L-tartrate, (h) (±)-Dimethyl tartrate.

*p*-Toluenesulfonic acid, Amberlyst 15 and Mont. K10 were investigated as catalysts in this study. Mont. K10 was found to be the most effective catalyst whereby the yields of acetals varied between 40 and 95%. The hydroxyl group is one of the most popular functional groups found in pharmaceuticals and natural products. For this reason, salicylaldehyde was chosen as a starting material in this study. The diols [18,19,20,21] used are commercially available materials as shown in Figure 1. 

## 2. Results and Discussion

### 2.1. Chemistry

**Scheme 1:**
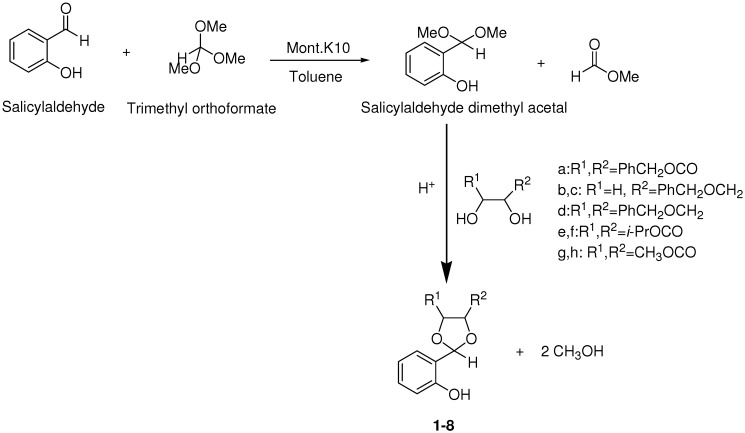
Synthesis of 1,3-dioxolanes.

At first**, **according to a conventional method**, **the direct synthesis of 1,3-dioxolanes from salicylaldehyde and diols a-h in the presence of Mont. K10 was investigated, however these reactions required long reaction times because the diols used in this study were sterically hindered. In our previous study [16], ketones with long alkyl chain were activated by trimethyl orthoformate (TMOF) to react with diols successfully in good yields and high enantiomeric excesses (>99% ee). In the literature [22,23,24], TMOF has been added in only a few studies as additive. Based on the previous study [16], salicylaldehyde was converted via TMOF to its more reactive dimethyl acetal (Scheme 1). This acetal participated then successfully in the acetalization reactions with chiral (compounds a,b,d,e,g) and racemic diols (compounds c,f,h) with shorter reaction times, good yields and high enantiomeric excesses (>99% ee). Chiral and racemic 1,3-dioxolanes **1**-**8** (Table 1) were obtained by the reaction of salicylaldehyde dimethyl acetal with chiral and racemic diols a-h. As shown in Table 1, salicylaldehyde can thus be efficiently transformed into their corresponding 1,3-dioxolanes in good to excellent isolated yields and excellent enantiomeric purity.

The effect of the structure of diols was investigated with regard to the overall yields of 1,3-dioxolanes. The benzylic groups with either ether or ester bonds are bound to the diols (compounds a, b, c, d), therefore these are more steric hindered than the other diols (compounds e, f, g, h). As shown in Table 1, when the steric hindered diols (compounds a,b,c,d) were used, the yields were found to be between 45%–61% (Entry 1-4). When the diols e, f, g, h were used, higher yields were obtained (88%–93%, Entries 5-8). The yields of 1,3-dioxolanes strongly depend on the structure of the diols. The overall yields decrease with increasing steric hindrance of the diols.

The diols a, b, d, e, g used in the synthesis of chiral 1,3-dioxolanes are enantiopure. Additionally, the chiral centers of these diols are not directly reacting. Due to these reasons, new chiral 1,3-dioxolanes of >99% ee values have been synthesized. Chiral HPLC of the new chiral 1,3-dioxolanes confirmed them to be highly enantiopure. This analysis yielded a single peak, indicating the presence of only one enantiomer in excellent enantiomeric excess. 

**Table 1:** Acetalization of salicylaldehyde with diols a-h by Montmorillonite K10 catalyst. ^a^

Entry	Diol	Product	Reaction Time (h)	ee ^b^ (%)	Yield ^c^ (%)
1	a	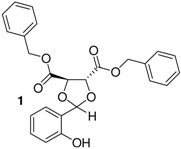	4	>99	45
2	b	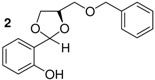	6	>99	53
3	c	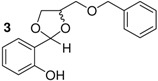	6	−	55
4	d	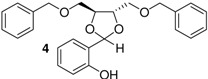	5	>99	61
5	e	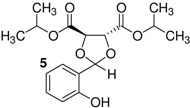	1	>99	88
6	f	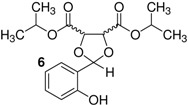	1	−	90
7	g	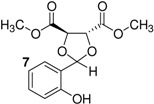	1	>99	92
8	h	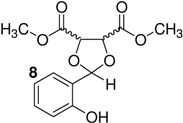	1	−	93

^a^ All reactions were carried out under Dean-Stark conditions; ^b^ The enantiomeric excesses were determined by HPLC using a Chiralcel OD column; ^c^ Yield of products isolated by silica gel column chromatography.

### 2.2. Biological Activity

All the synthesized derivatives **1-8** were investigated for their *in vitro* antibacterial activities against three Gram-positive (*Staphylococcus aureus* ATCC 29213, *Staphylococcus epidermidis* ATCC 12228, *Enterococcus faecalis* ATCC 29212) and four Gram-negative bacteria (*Pseudomonas aeruginosa* ATCC 27853, *Escherichia coli* ATCC 25922, *Klebsiella pneumoniae* ATCC 4352, *Proteus mirabilis* ATCC 14153). The antifungal activity was tested against a yeast *Candida albicans* ATCC 10231. All the biological results of the tested compounds are given in Table 2.

As seen in Table 2, all of the new compounds, except **7**, possessed excellent activity against *S. aureus* with MIC values of between 625–1250 µg/mL. The synthesized compounds, except **3**, also showed excellent antibacterial activity against *S. epidermidis*. In addition, some members of the 1,3-dioxolane derivatives, like **4,6,8**, displayed perfect antibacterial activity against *P. aeruginosa*. Among the tested compounds, only **4** showed perfect activity with the MIC value of 625 µg/mL against *E. faecalis*. On the other hand, none of the new compounds exhibited antibacterial activity against *E. coli*, *K. pneumoniae* and *P. mirabilis*. Antifungal screening result has indicated that all of the new compounds, except **1**, showed significant antifungal activity against *C. albicans*.

**Table 2:** *In vitro* antibacterial and antifungal activity of 1,3-Dioxolane derivatives.

**Microorganisms**	**MIC Values** (µg/mL)								
		1	2	3	4	5	6	7	8
***S. aureus***		1250	1250	1250	312.5	625	625	-	1250
***S. epidermidis***		625	1250	-	625	1250	625	1250	625
***E. faecalis***		-	-	-	625	-	-	-	-
***P. aeruginosa***		-	-	-	625	-	625	-	625
***E. coli***		-	-.	-	-	-	-	-	-
***K. pneumoniae***		-	-	-	-	-	-	-	-
***P. mirabilis***		-	-	-	-	-	-	-	-
***C. albicans***		-	312.5	78.12	312.5	156.25	156.25	312.5	312.5

The comparative biological activity effects of the enantiomeric and racemic structured of the compounds **2,3,5,6,7,8** mentioned in this work was further investigated. While the chiral compound **2 **showed antibacterial activity against *S. epidermidis*, its racemic form **3** has not shown *in vitro* activity against this species. The chiral compounds **5 **and **7** possessed biological no activity at all against *P. aeruginosa*. In contrast, their corresponding racemic compounds **6** and **8** showed perfect antibacterial activity against *P. aeruginosa*. While the chiral compound **7** is biological inactive against *S. aureus* its racemic compound **8** is biologically active. However, the both forms, the chiral **5** and its racemic **6**, played significant activity against *S. aureus*. According to these results, a general conclusion cannot be drawn in terms of biological activity on whether enantiomerically pure compounds and their corresponding racemic compounds have antibacterial or antifungal activities. This strategy can be explained with a key-lock model.

## 3. Experimental

### 3.1. General

All reagents were obtained from commercial suppliers and used as provided without further purification. Toluene was dried over sodium. The reactions were monitored by TLC (Merck 60 F-254). Column chromatography was performed on silica gel 60 (70–230 mesh). ^1^H- and ^13^C-NMR Spectra: Varian 400; chemical shifts (*δ*) reported in ppm relative to TMS as internal standard; coupling constants (*J*) in Hz. Mass spectra (ESI) were recorded on a Thermo Finnigan LCQ Advantage Max spectrometer. Infrared (IR) spectra were recorded on a Mattson 1000 series spectrometer; absorptions given in cm^-1^. Melting points (uncorrected) were taken on a Büchi B-540 melting point apparatus. Optical rotations were measured *on an Optical Activity AA-55* digital polarimeter and were the average of more than five measurements. Enantiomeric purities of 1,3-dioxolanes were determined with a Shimadzu/DGU-20A_5_ HPLC apparatus equipped with a Daicel Chiralcel OD column chiral.

### 3.2. General Procedure for the Synthesis of 1,3-Dioxolanes ***1-8***

A mixture of salicylaldehyde (0.122 g, 1.0 mmol), trimethyl orthoformate (0.11 mL, 1.0 mmol), montmorillonite K10 (300 mg) and sodium dried toluene (20.0 mL) were placed in a round bottom flask fitted with a Dean-Stark apparatus to stir for 1h, then diol (2.0 mmol) was added and the mixture refluxed for the indicated times (Table 1) with removal of the methanol formed. The progress of the reaction was monitored by TLC. After cooling, the catalyst was removed by filtration. The reaction mixture was washed with solid NaHCO_3_ and water. The organic layer was dried (MgSO_4_) and the solvent was evoporated under reduced pressure. The product was purified by flash chromatography (silica gel, hexane/ethyl acetate as an eluent) to give the 1,3-dioxolane in excellent yield.

*Bis(benzyl)(4R,5R)-2-(2-hydroxyphenyl)-1,3-dioxolane-4,5-dicarboxylate* (**1**). White crystals. M.p = 104–105 °C. Yield 45%. >99% ee. [α]^20^_D_ = −40 (*c* 1,CHCl_3_). IR (KBr): 3400, 3060, 2946, 1754, 1483, 1402, 1239, 1104, 752, 698. ^1^H-NMR (CDCl_3_): 4.79 (d, *J* = 4.3, 1H); 4.91 (d, *J* = 3.9, 1H); 5.15 (d, 2H); 5.20 (d, 2H); 6.07 (s, 1H); 7.18–7.47 (m, 14H); 7.92 (s, 1H). ^13^C-NMR (CDCl_3_): 66.54; 73.33; 76.58; 105.89; 126.19; 128.92; 133.95; 134.40; 167.79; 155.32. ESI-MS: 434.92 ([M]^+^). Anal.Calc. for C_25_H_22_O_7_: C, 69.12; H, 5.10. Found: C, 69.22; H, 5.06. HPLC analysis: mobile phase *iso*-PrOH/hexane: 10/90, 35 °C, flow rate: 1.0 mL/min, wavelength: 210 nm; *t*_R_ (retention time): 15.324 min.

*2-{(4R)-4[(benzyloxy)methyl]-1,3-dioxolane-2-yl}phenol* (**2**). Colorless oil. Yield 53%. >99% ee. [α]^20^_D_ = −12 (*c* 1,CHCl_3_). IR (KBr): 3407, 3055, 2919, 1646,1510, 1266, 1077, 752, 725. ^1^H-NMR (CDCl_3_): 3.39–3.62 (m, 2H); 3.80–4.16 (m, 2H); 4.28–4.37 (m, 1H); 4.48 (m, 2H); 5.66 (s, 1H); 6.70–7.29 (m, 9H); 7.85 (s, 1H). ^13^C-NMR (CDCl_3_): 67.41; 70.31; 73.86; 75.24; 117.40; 131.43; 119.97; 137.39; 156.46. ESI-MS: 286.18 ([M]^+^). Anal.Calc. for C_17_H_18_O_4_: C, 71.31; H, 6.34. Found: C, 71.43; H, 5.31. HPLC analysis: mobile phase *iso*-PrOH/hexane: 10/90, 35 °C, flow rate: 1.0 mL/min, wavelength: 210 nm; *t*_R_ (retention time): 17.096 min.

*2-{4[(benzyloxy)methyl]-1,3-dioxolane-2-yl}phenol* (**3**). This compound was synthesized by the same method described above reaction using salicylaldehyde and diol c. The spectroscopic datas (IR, NMR (^1^H, ^13^C), MS, elemental analysis) were equal to those of compound **2**.

*2-[(4S,5S)-4,5-bis(benzyloxymethyl)-1,3-dioxolane-2-yl]phenol* (**4**). Colorless oil. Yield 81%. >99% ee. [α]^20^_D_ = −10 (*c* 1,CHCl_3_). IR (KBr): 3407, 3082, 3055, 2892, 1646, 1592, 1483, 1375, 1266, 1104, 752, 725. ^1^H-NMR (CDCl_3_): 3.48 (d, *J* = 2.44, 2H); 3.69 (d, *J* = 2.44, 2H); 4.50 (quartet, *J* = 7.81, 2H); 4.62 (s, 2H); 4.64 (s, 2H); 5.90 (s, 1H); 6.75–7.29 (m, 14H); 7.86 (s, 1H). ^13^C-NMR (CDCl_3_): 67.53; 69.12; 75.49; 77.04; 105.20; 116.62; 130.11; 119.09; 136.21; 136.79; 155.21. ESI-MS: 406.92 ([M]^+^). Anal.Calc. for C_25_H_26_O_5_: C, 73.87; H, 6.45. Found: C, 73.22; H, 5.39. HPLC analysis: mobile phase *iso*-PrOH/hexane: 10/90, 35 °C, flow rate: 1.0 mL/min, wavelength: 210 nm; *t*_R_ (retention time): 15.824 min.

*Diisopropyl (4R-5R)-2-(2-hydroxyphenyl)-1,3-dioxolane-4,5-dicarboxylate* (**5**). Colorless oil. Yield 88%. >99% ee. [α]^20^_D_ = −56 (*c* 1,CHCl_3_). IR (KBr): 3334, 3050, 2973, 1754, 1239, 1104, 752, 725. ^1^H-NMR (CDCl_3_): 1.24 (d, *J* = 6.35, 6H,); 4.62 (d, 1H); 4.69 (d, 1H); 5.06-5.11 (heptet, *J* = 3.42, 2H); 6.02 (s, 1H); 6.77–7.47 (m, 5H); 8.11 (s, 1H). ^13^C-NMR (CDCl_3_): 20.49; 20.66; 69.24; 70.02; 75.65; 76.34; 103.50; 117.08; 118.44; 129.11; 130.79; 155.16; 167.69; 168.95. ESI-MS: 361.83 ([M+Na]^+^). Anal.Calc. for C_17_H_22_O_7_: C, 60.35; H, 6.55. Found: C, 61.30; H, 6.65. HPLC analysis: mobile phase *iso*-PrOH/hexane: 5/95, 35 °C, flow rate: 1.0 mL/min, wavelength: 210 nm; *t*_R_ (retention time): 7.522 min.

*Diisopropyl 2-(2-Hydroxyphenyl)-1,3-dioxolane-4,5-dicarboxylate* (**6**). This compound was synthesized by the same method above from salicylaldehyde and f. The spectroscopic datas (IR, NMR (^1^H, ^13^C), MS, elemental analysis) were equal to those of compound **5**.

*Dimethyl (4R-5R)-2-(2-Hydroxyphenyl)-1,3-dioxolane-4,5-dicarboxylate* (**7**). White crystal. M.p= 94–95 °C. Yield 92%. >99% ee. [α]^20^_D_ = −80 (*c* 1,CHCl_3_). IR (KBr): 3334, 3050, 2973, 1754, 1239, 1104, 752, 725. ^1^H-NMR (CDCl_3_): 3.81 (s, 6H); 4.75 (d, *J* = 3.4, 1H); 4.81 (d, *J* = 3.4**, **1H); 6.03 (s, 1H); 6.79–7.26 (m, 4H); 7.98 (s,1H). ^13^C-NMR (CDCl_3_): 52.02; 52.47; 75.25; 76.02; 107.43; 116.69; 117.15; 118.56; 129.17; 130.93; 155.07; 168.46; 169.82. ESI-MS: 282.78 ([M]^+^). Anal.Calc. for C_13_H_14_O_7_: C, 55.32; H, 5.00. Found: C, 55.51; H, 4.98. HPLC analysis: mobile phase *iso*-PrOH/hexane: 5/95, 35 °C, flow rate: 1.0 mL/min, wavelength: 210 nm; *t*_R_ (retention time): 7.572 min.

*Dimethyl 2-(2-Hydroxyphenyl)-1,3-dioxolane-4,5-dicarboxylate* (**8**). This compound was synthesized by the same method described above from salicylaldehyde and compound g. The spectroscopic datas (IR, NMR (^1^H, ^13^C), MS, elemental analysis) were equal to those of **7**.

### 3.3. Biological Assays

*In vitro* antibacterial activities of new compounds against *S. aureus* ATCC 29213, *S. epidermidis* ATCC 12228, *E. faecalis* 29212, *P. aeruginosa* ATCC 27853, *E. coli* ATCC 25922, *K. pneumoniae* ATCC 4352, *P. mirabilis* ATCC 14153 and antifungal activities against *C. albicans* ATCC 10231 were investigated. Minimum inhibitory concentrations (MICs) of compounds were determined by microbroth dilution technique as described by the Clinical and Laboratory Standards Institute [25,26]. Serial two fold dilutions ranging from 5000 to 4.8 µg/mL were prepared in Mueller-Hinton broth (MHB) for bacteria and RPMI-1640 medium for yeast. Each well was inoculated with 50 µL of a 4 to 6 hour broth culture that gave a final concentration of 5 × 10^5^ cfu/mL for bacteria and 5 × 10^3^ cfu/mL for yeast in the test tray. The trays were covered and placed in plastic bags to prevent evaporation. The trays containing MHB were incubated at 35 °C for 18–20 h, those containing RPMI-1640 medium at 35 °C for 46–50 h. The MIC was defined as the lowest concentration of compound producing complete inhibition of visible growth. Amikacin and fluconazole were used as reference antibiotics for bacteria and yeast, respectively. The MIC values of the amikacin and fluconazole were within the accuracy range in CLSI throughout the study [27]. The MIC values of the compounds are given in Table 2.

## 4. Conclusions

We have synthesized some new derivatives of chiral and racemic 1,3-dioxolanes. All the compounds were tested as new antibacterial and antifungal agents. It is important to note that the new chiral and racemic 1,3-dioxolanes have shown excellent antibacterial and antifungal activities. These compounds could be evaluated as bioactive agents in pharmaceutical industry.
